# Changes in female function and autonomous selfing across floral lifespan interact to drive variation in the cost of selfing

**DOI:** 10.1002/ajb2.1816

**Published:** 2022-03-27

**Authors:** Rachel B. Spigler, Rossana Maguiña

**Affiliations:** ^1^ Department of Biology Temple University 1900 N 12th St. Philadelphia PA 19122 USA; ^2^ Present address: Rossana Maguiña, Ecology and Evolutionary Biology Department University of California Santa Cruz 130 McAllister Way Santa Cruz CA 95060 USA

**Keywords:** delayed selfing, dichogamy, floral senescence, flower age, Gentianaceae, pollinator limitation, protandry, reproductive assurance, stigmatic receptivity, timing of selfing

## Abstract

**Premise:**

Morphological and developmental changes as flowers age can impact patterns of mating. At the same time, direct or indirect costs of floral longevity can alter their fitness outcomes. This influence has been less appreciated, particularly with respect to the timing of selfing. We investigated changes in stigma events, autonomous selfing, outcross seed set capacity, and autofertility—a measure representing the potential for reproductive assurance—across floral lifespan in the mixed‐mating biennial *Sabatia angularis*.

**Methods:**

We examined stigma morphology and receptivity, autonomous self‐pollen deposition, and seed number and size under autonomous self‐pollination and hand outcross‐pollination for flowers of different ages, from 1 d of female phase until 14 d. We compared autonomous seed production to maximal outcross seed production at each flower age to calculate an index of autofertility.

**Results:**

The stigmatic lobes begin to untwist 1 d post anthesis. They progressively open, sextend, coil, and increase in receptivity, peaking or saturating at 8–11 d, depending on the measure. Autonomous seed production can occur early, but on average remains low until 6 d, when it doubles. In contrast, outcross seed number and size start out high, then decline precipitously. Consequently, autofertility increases steeply across floral lifespan.

**Conclusions:**

Changes in stigma morphology and receptivity, timing of autonomous self‐pollen deposition, and floral senescence can interact to influence the relative benefit of autonomous selfing across floral lifespan. Our work highlights the interplay between evolution of floral longevity and the mating system, with implications for the maintenance of mixed mating in *S. angularis*.

Floral longevity, defined as the amount of time a flower remains open and functional, varies remarkably across species, from no more than a single day to more than a month (Primack, 1985; Ashman and Schoen, 1996). This definition, however, belies that the capacity for pollen export, import, and seed production can change across floral lifespan. These changes may occur as part of genetically programmed floral development, flower ageing and senescence, external conditions (e.g., temperature, pollinator availability), or plastic responses to external and internal (e.g., mating experience, pollen receipt) conditions (Marshall et al., 2010). Understanding whether and how these changes play out across floral lifespan is important for understanding opportunities for intraspecific pollen competition, sexual interference, and self‐fertilization, all of which influence plant mating patterns, fitness, and floral evolution.

In particular, morphology of the stigma and style and stigmatic receptivity can change over the course of floral lifespan. Many species exhibit style elongation, stigmatic or style curvature, or stigma movements, including closure and reopening (Ruan and Teixeira da Silva, 2011; Goodwillie and Weber, 2018). In some species, changes in stigma morphology are induced by pollination (e.g., Waser and Fugate, 1986; Fetscher and Kohn, 1999), but in others, they may represent “active” movements, i.e., by the plant alone, following a predictable pattern over floral lifespan (e.g., Kalisz et al., 1999; Freitas and Sazima, 2009; Ruan et al., 2010). A number of hypotheses have been put forward to explain the adaptive significance of these developmental patterns, including avoidance of (or a reduction in) sexual interference, promotion of outcrossing, and stalling any potential selfing (Lloyd and Webb, 1986; Fetscher, 2001; Barrett, 2002; and reviewed by Ruan and Teixeira da Silva, 2011). For example, some active movements decrease the distance between the stigma and anthers, facilitating the potential for autonomous selfing later in floral lifespan, after opportunities for outcrossing are exhausted. In other species, changes in stigmatic receptivity functionally create temporal separation of male and female phase. Delayed onset of receptivity causes protandry, whereas protogyny may arise via a decline in receptivity before anthers dehisce. In addition to reducing sexual interference, delayed onset of stigmatic receptivity may allow for a greater number and/or diversity of pollen grains when pollen is allowed to accumulate, promoting greater pollen competition and/or mate choice (Murdy and Carter, 1987; Dahl and Fredrikson, 1996; Herrero, 2003; Lankinen et al., 2007; but see Galen et al., 1986).

For self‐compatible species, internal changes within flowers interact with pollinator availability and behavior to determine whether and when self‐fertilization occurs during floral lifespan. The timing of selfing is a critical factor influencing mating system evolution, with the potential to compound or alleviate its costs. Indeed, Lloyd (1992) and Lloyd and Schoen (1992) pointed out that not all modes of selfing are created equally and explicitly considered for autonomous selfing, which has the potential to provide reproductive assurance, the implications of “prior selfing” that occurs early in floral lifespan before opportunities for outcross pollen deposition, “competing selfing” that occurs during the period of outcross pollen deposition, and “delayed selfing” that occurs in older flowers, once outcross opportunities have passed. Despite possible benefits, prior and competing selfing can reduce the amount of pollen available for outcrossing (pollen discounting; Holsinger et al., 1984; Lloyd, 1992) and usurp ovules that would otherwise be fertilized by outcross pollen, lowering fitness for species with high inbreeding depression (seed discounting; Lloyd, 1992; Herlihy and Eckert, 2002; Eckert et al., 2006). Moreover, relatively early autonomous selfing could further reduce or even preclude outcross pollen export or import if it triggers floral senescence and reduces floral longevity (Weber and Goodwillie, 2013; Spigler, 2017). In contrast, delayed selfing is typically considered the “best of both worlds”, enabling plants to prioritize outcrossing and self when outcrossing fails (Becerra and Lloyd, 1992; Lloyd, 1992; Kalisz and Vogler, 2003; Vaughton and Ramsey, 2010; Goodwillie and Weber, 2018). In this case, even under high inbreeding depression, selfing can provide “reproductive assurance”, because it is still better than complete reproductive failure. Delayed selfing can arise from developmental changes within flowers, including incomplete dichogamy, movement herkogamy, or progressive stigma curvature, for example (reviewed by Goodwillie and Weber, 2018).

The quality of each flower can also degrade over its lifespan. One potential major consequence is a decline in seed production with age (e.g., Jakobsen and Martens, 1994; Petanidou et al., 2001; Castro et al., 2008; Hildesheim et al., 2019), possibly caused by poorer pollen tube development in the style, decreased stigmatic receptivity, and decreased ovule viability associated with senescence or caused by increased seed abortion related to floral maintenance costs. Pollen quality can also decline with age (Smith‐Huerta and Vasek, 1984; Dafni and Firmage, 2000; Petanidou et al., 2001; Malagon et al., 2019). These changes can influence both mating success and patterns, and even the cost of selfing. For one, age‐related seed declines put a premium on earlier‐arriving pollen, intensifying the priority effect (e.g., Spira et al., 1996; Burkhardt et al., 2009). Second, declines in seed production with flower age represents a cost, or at least a reduced benefit, of plant investment in floral maintenance (Ashman and Schoen, 1997; Abdala‐Roberts et al., 2007) and should strongly influence the evolution of optimal floral longevity (Ashman and Schoen, 1994; Schoen and Ashman, 1995). Finally, changes in maximum seed quantity or quality with age should also interact with the timing of selfing to determine its fitness costs (Hildesheim et al., 2019) and ability to provide reproductive assurance (i.e., autofertility). In fact, autofertility explicitly compares autonomous selfing ability relative to the maximum seed production that could potentially be achieved and is considered a key parameter in mating system evolution (Lloyd and Schoen, 1992; Eckert et al., 2010; Razanajatovo et al., 2019).

In this paper, we examine changes in flower development with flower age in the self‐compatible plant *Sabatia angularis* (L.) Pursh (Gentianaceae) and consider the potential impacts on mating patterns. *Sabatia angularis* presents an excellent study system for understanding the interplay between the evolution of floral longevity and the mating system. The species has relatively long‐lived, protandrous flowers, with floral lifespan ranging from ~4 d up to ~20 d depending on pollination conditions (Spigler, 2017). Prior work has demonstrated that autonomous selfing can provide reproductive assurance (Spigler, 2018), but the mechanism and timing are unknown. Given high inbreeding depression in at least some populations (Dudash, 1990; Spigler et al., 2017), the timing of selfing will be a critical determinant of the true cost of selfing in this species. We perform a set of complementary experiments to address the following questions. (1) Do stigma morphology and receptivity vary predictably with flower age in *S. angularis*? (2) What is the timing of autonomous self‐pollen deposition, and how does autonomous seed production vary with flower age? (3) Do seed quantity and/or quality under outcross‐pollination depend on flower age? And finally, (4) does autofertility vary with flower age? The answers to these questions can cast light onto the factors shaping optimal floral longevity and the stability of mixed mating.

## MATERIALS AND METHODS

### Study species

*Sabatia angularis* is a biennial herb native to the eastern United States and Canada. Seeds disperse in the fall, germinate in the spring, and develop into rosettes, which overwinter and bolt the following spring. From July to August, plants produce displays of showy, pink, pollen‐rewarding flowers that ripen into dry, dehiscent capsules. Individual flowers contain ~1000–1200 ovules and are protandrous, although protandry can be incomplete, depending on rates of pollen removal. The stigma is bilobed; the lobes are tightly wound at anthesis and begin to unravel and expose the receptive surface approximately 1 d later (mean 1.15 d ± 0.25 SD) (Spigler and Woodard, 2019). Perry (1971) noted that the stigmatic lobes of many *Sabatia* species become coiled at maturity, though the timing of this event during floral lifespan is not documented. The species is self‐compatible and capable of autonomous selfing (Spigler, 2018), with populations varying from mixed mating to highly outcrossing (Spigler et al., 2010). The mechanism and timing of autonomous self‐pollination is unclear. Self‐pollen deposition could occur early in floral lifespan due to incomplete protandry, accumulate gradually or stochastically across floral lifespan when pollen removal rates are low, or be related to previously observed stigma coiling.

Study plants for each of the experiments described below were grown from open‐pollinated seed originating from six wild populations across the serpentine grasslands of southeastern Pennsylvania, United States (populations F9, HM, MB, PH, SB2, and UB2 in Emel et al., 2017 and Spigler, 2018). We note that we included plants from multiple populations as our goal was to evaluate average patterns for the species, not to test for population variation in our traits of interest. All experiments were carried out in Temple University's Plant Facility under controlled, pollinator‐free conditions. Up to 35 plants were assigned to each experiment; final sample sizes varied due to mortality (see below).

### Stigmatic morphology and receptivity across floral lifespan

**Figure 1:**
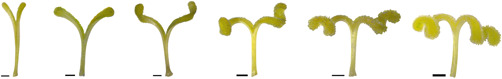
Time sequence of *Sabatia angularis* stigmas. Stigma lobes are wrapped upon anthesis and then begin to unravel, expand, and curl across floral lifespan. Figure shows pictures of stigmas sampled across female phase throughout floral lifespan (from left to right: days 1, 2, 3, 5, 7, and 12). Scale bars = 1 mm

We collected stigmas on different days (day 1, 2, 3, 4, 6, 8, 10, 12, or 14) of female phase on each of 31 plants (originating from four of the six populations, *N* = 7–9 plants per population) to evaluate stigmatic development across floral lifespan. Our goal was to get at least one replicate per age per plant and up to two for plants that had enough flowers. This resulted in a total of 332 flowers (average 10.4 ± 2.28 SD per plant), with treatments randomly assigned to flower buds before opening. Each flower was emasculated at the bud stage to prevent potential autonomous self‐pollination and subsequent pollination‐induced stigma wilting (Spigler, 2017). To emasculate the flower, we used forceps to gently open large, pink flower buds (likely to open within 1–2 d, based on Spigler, 2017) just enough to access the undehisced anthers and remove them with the forceps. Prior work has demonstrated the removal does not damage petals, reduce corolla lifespan, or have a negative impact on seed production flowers (Spigler, 2017, 2018). Day 1 of female phase was defined as the date that the stigma lobes begin to unravel and expose the receptive surface and was previously found to start nearly invariably 1 d after anthesis, i.e., 2‐d‐old flowers (Spigler and Woodard, 2019). To test stigmatic receptivity, we collected stigmas on the appropriate assigned day with forceps by pinching the style above where it meets the ovary and then immediately placing them under a dissecting microscope in a benzidine‐H_2_O_2_ solution following Dafni (1992). This solution causes stigmas to turn blue and produce bubbles when there is enzymatic activity. After stigmas were immersed in the solution for 60 s, we captured a digital image. We scored stigma receptivity according to an ordinal scale representing the amount of bubbling that occurred in the benzidine solution on the stigmatic surface and that we found to be repeatable (0 = no bubbling; 1 = few bubbles; 2 = many bubbles). We confirmed that bubbling coincided with blue staining of the stigmatic surface and was not associated with the torn end of the style where it was removed from the flower. The following measurements were later made from the digital images using ImageJ software (Schneider et al., 2012): angle of stigma opening, measured as the angle (degrees) between the two stigma lobes; stigma lobe extension, measured as the distance from the style to the end of each stigma lobe at a 90‐degree angle from the style (averaged across lobes); and the number of coils per stigma lobe (maximum of the two lobes) to represent the degree of stigma coiling (see Figure 1 and Appendix S1).

### Timing of autonomous selfing

We evaluated the timing of autonomous selfing on 32 plants (originating from five populations, N = 4–9 plants per population). On each plant, we tagged flower buds before they opened and randomly assigned each to have their anthers removed on either day 1, 2, 3, 4, 6, 8, 10, or 12 of female phase, with two replicates per treatment per plant, except for two plants that did not have enough flowers (total *N* = 497 flowers). *Sabatia angularis* flowers are arranged on a compound, cymose inflorescence with a determinate axis. The primary position is the terminal flower of the main axis. We focused on buds located at terminal positions of lateral branches (i.e., secondary positions) to control for potential differences in ovule number across floral positions (e.g., Diggle, 1995; Guitián and Navarro, 1996). In some cases, we needed to sample at tertiary or quaternary positions. We recorded position in all cases. Before anther removal, we wet anthers with a cotton swab to avoid incidental pollination and then removed anthers by pinching the filament of each stamen with forceps. Removal of anthers on a given day means that any resultant seed must have resulted from autonomous self‐pollen deposition on or before the day of removal. We used anther removal rather than stigma removal because prior work already demonstrated anther removal does not impact seed production; seed set from hand pollination was the same between emasculated and intact flowers (Spigler, 2018). Moreover, this approach allows direct comparison to seed production from hand outcross‐pollinations across floral lifespan (see below). On a subset of the 32 plants (*N* = 15 plants), we collected a single stigma per treatment using forceps once stigmas were wilted to examine self‐pollen deposition rates. We mounted stigmas onto a microscope slide after softening with NaOH and examined slides on a light microscope to count all pollen grains per stigma. We then collected ripe fruits before opening and captured digital images of seeds. We counted seeds and estimated average seed size (diameter in millimeters) per fruit using ImageJ software. We considered seed size as a metric of seed quality. Seed size is a well‐known indicator of seedling performance both across (Westoby et al., 1996) and within species (e.g., Stanton, 1984). Moreover, data from this experiment do not support a seed size‐number trade‐off (*r* = 0.13, *P* = 0.01).

### Maximum seed production across floral lifespan

We quantified seed production resulting from outcross hand pollination across floral lifespan on a different set of plants (21 plants originating from two populations, *N* = 10–11 plants per population). On each plant, we randomly assigned flower buds to be pollinated with outcross pollen (“maximum outcross‐pollination”) on either day 1, 2, 3, 4, 6, 8, 10, 12, or 14 of female phase. Treatments were replicated twice per plant, except when there were not enough flowers (total *N* = 497 flowers), and we recorded position for each bud as indicated above. To prevent self‐pollination, we emasculated focal flowers in the bud stage using the same procedure as described above. On the assigned day, we rubbed dehisced anthers collected from an outcross donor onto the exposed surface of the stigma until the stigmas were visibly covered with the yellow pollen. A single outcross donor was used per flower, but different donors were used across flowers. Donors were haphazardly chosen, unrelated, non‐focal plants. We collected ripe fruits prior to capsule dehiscence and determined seed number and average seed size (diameter in millimeters) from digital images as outlined for autonomous seed set. For this experiment as well, we did not find a trade‐off; seed size and number were positively related (*r* = 0.59, *P* < 0.0001).

### Statistical analyses

All statistical analyses were generated using SAS software (version 9.3, SAS Institute, Cary, NC, USA). We used general linear mixed models (GLM, proc mixed) to investigate whether the following response variables changed as a function of flower age: angle of opening, stigma lobe extension, self‐pollen grain deposition (log‐transformed), seed number and average seed size per fruit via autonomous self‐pollination and maximum outcross‐pollination. In each model, flower age was treated as a continuous predictor variable, and we included plant identity (nested within population) as a random effect to account for measurements on multiple flowers per plant. We also considered a random population effect and, where relevant based on scatterplots, heterogeneity of variance among populations. Based on scatterplots of the data, we also considered a quadratic flower age term and retained where *P* ≤ 0.05. Because stigmatic receptivity was scored on an ordinal scale (0, 1, or 2) and data for stigmatic coiling (using the maximum number found per lobe) in our data set were limited to values of 0, 1, and 2, for these response variables we used generalized linear mixed models with a multinomial distribution and cumulative logit link function (proc glimmix), including the same fixed and random predictor variables used in the GLMs. For each response variable, we compared models with and without random effects and retained random effects where ΔAIC > 2. Where ΔAIC < 2 for the top models, we present the simplest model (Appendix S2). For seed production (both autonomous and hand‐pollinated), we also included flower position (values 2, 3, or 4) as a continuous covariate and retained where *P* ≤ 0.05.

Finally, we calculated an index of autofertility at 1, 2, 3, 4, 6, 8, 10, 12 d of female phase. Autofertility can be calculated as autonomous seed production divided by seed production under hand pollination (e.g., Eckert et al., 2010; Razanajatovo et al., 2019). Because we quantified autonomous and maximum outcross‐pollination on different sets of plants, we calculated one autofertility index per day based on mean autonomous seed production and maximum outcross‐pollination per day of female phase. Thus, the relationship between autofertility and flower age in our study does not take into account individual or population variation and is meant to serve as a species‐level estimate. We used general linear regression (proc glm) to evaluate whether autofertility varied with flower age, considering a polynomial relationship with higher level terms for flower age.

## RESULTS

**Figure 2:**
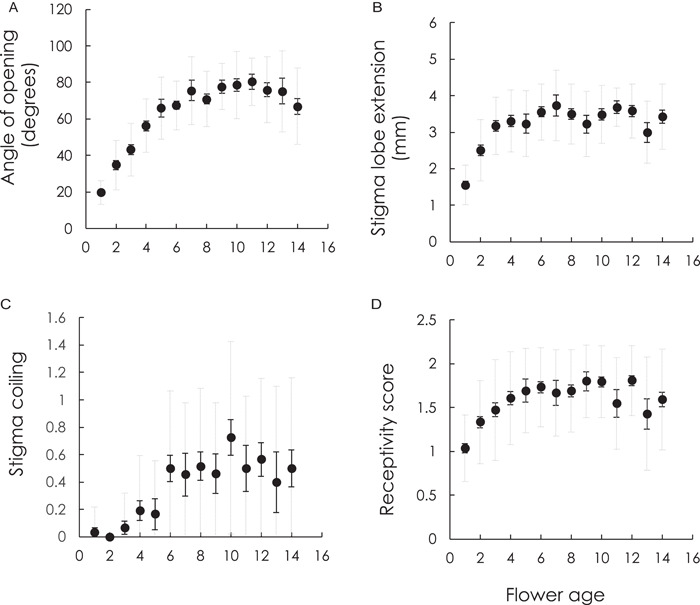
Change in stigma morphology and receptivity across floral lifespan. (A) Angle of opening between stigma lobes. (B) Extension of stigma lobes. (C) Stigma coiling. (D) Stigmatic receptivity. Means per day are presented with SE (solid error bars) and SD (dotted gray error bars) and reported in Appendix S3. Note the *x*‐axis represents day of female phase, which begins ~1 d post anthesis

Stigma morphology and receptivity follow a predictable sequence across floral lifespan (Figure 1). At 1 d post anthesis, the stigma lobes begin to unravel. As the flower ages, the lobes continue to separate and begin to coil, leading to changes in the size and shape of the exposed stigmatic surface. Mean angle of opening, stigma lobe extension, coiling, and receptivity as measured by the peroxidase all significantly increase across flower age (Table 1, Figure 2). Models for all these traits included significant quadratic terms, indicating they either peak or saturate before the end of floral lifespan (predicted peak ~8–11 d, depending on the trait).

**Table 1:** Mixed‐model results for the fixed effect^a^ of flower age on stigma morphology and receptivity, as measured by the peroxidase test, across floral lifespan

	Angle of opening				Stigma extension				Stigma coiling				Receptivity			
Flower age	df	Estimate	*F*	*P*	df	Estimate	*F*	*P*	df	Estimate	*F*	*P*	df	Estimate	*F*	*P*
Linear	1, 286	13.5	377.88	**<0.0001**	1, 287	0.45	119.22	**<0.0001**	1, 280	1.97	79.41	**<0.0001**	1, 525	0.78	60.57	**<0.0001**
Quadratic	1, 286	–0.66	203.07	**<0.0001**	1, 287	–0.02	77.86	**<0.0001**	1, 280	–0.095	57.9	**<0.0001**	1, 525	–0.04	43.07	**<0.0001**

^a^ See Appendix S3 for a table of random effects for each model.

**Figure 3:**
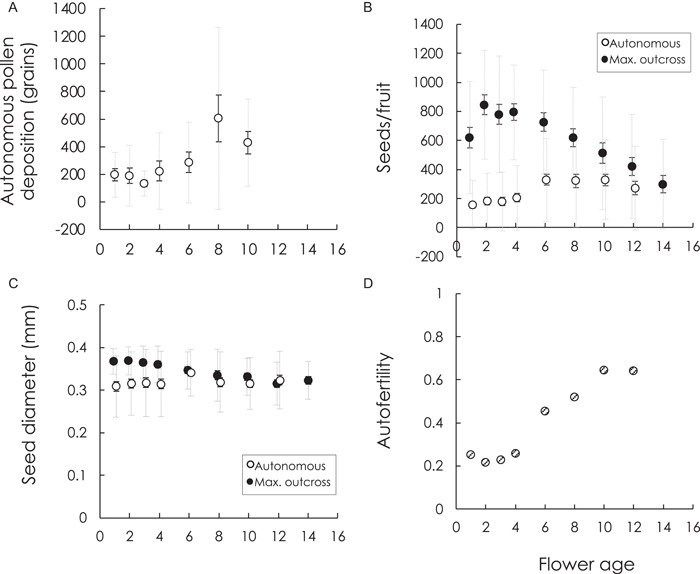
Changes in autonomous selfing and maximum seed production and seed size across floral lifespan and their consequences for autofertility. (A) Autonomous pollen deposition. (B) Seeds per fruit under autonomous self‐pollination (open circles) and hand outcross‐pollination (filled circles). (C) Diameter of seeds produced by autonomous self‐pollination (open circles) and hand outcross‐pollination (filled circles). (D) Autofertility, measured as the ratio of seed number by autonomous selfing to maximum outcross‐pollination. Means per day are presented with SE (solid error bars) and SD (dotted gray error bars) and reported in Appendix S3. Note the x‐axis represents day of female phase, which begins ~1 d post anthesis. For B and C, data points for autonomous selfing and hand outcross‐pollination are offset by ±0.1 d on the *x*‐axis to avoid overlap of error bars

Autonomous self‐pollen deposition can start early, though the number of grains is low relative to the number of ovules per flower (Figure 3A). Self‐pollen deposition continues to increase across floral lifespan (Table 2). Mean daily autonomous seed production also increases with flower age; however, this relationship was best modelled as a quadratic, indicating that it peaks or saturates before wilting (Table 2, Figure 3B). Mean autonomous self‐pollen deposition and autonomous seed number for each flower age were correlated (*r* = 0.44, *N* = 103, *P* < 0.0001), highly so when based on means per day (*r* = 0.83, *N* = 7, *P* = 0.02). Mean size of autonomously selfed seeds, however, did not change with flower age (Table 1, Figure 3C).

Both mean seed number per fruit and seed size resulting from maximum outcross‐pollination changed with flower age (Table 1). Seed number followed a quadratic relationship (Figure 3B), increasing from day 1 to day 2, reaching its predicted maximum at ~3 d, and then steadily declining. We note that flower position also influenced maximum outcross seed number per fruit (*F*_1,240_ = 7.91, *P* = 0.005). As expected, seed number per fruit decreased as position increased; i.e., it was lower in later opening flowers occurring at higher level branch points in the cymose inflorescence. Outcrossed seed size declined linearly with flower age (Figure 3C).

By the end of floral lifespan, seed number and size resulting from hand outcross‐pollination and autonomous self‐pollination converged (Figure 3B, C). Consequently, the relative difference decreases and the autofertility index increases across floral lifespan (linear model *F* = 79.29, *P* = 0.0001, *R* ^2^ = 0.93; cubic model *F* = 85.12, *P* = 0.0004, *R* ^2^ = 0.98) (Figure 3D).

## DISCUSSION

### Form, function, and the timing of selfing

Developmental changes across floral lifespan occur for many species (Marshall et al., 2010). We showed predictable changes in stigma morphology and receptivity with flower age in *S. angularis*. The tightly wound stigma begins to unravel 1d after anthesis, but at this time point only slightly so, gradually unraveling, extending, coiling and becoming more receptive across floral lifespan. These changes play out over for over a week, either reaching a peak or remaining steady for the remainder of floral lifespan (Figure 2). From our study, we cannot assess fitness consequences, but beyond the general benefits of protandry (see, e.g., Lloyd and Yates, 1982; Lloyd and Webb, 1986) the gradual opening and increasing receptivity of the stigma are consistent with multiple, non‐mutually exclusive hypotheses. For one, continuous schedules of receptivity rather than a restricted period allow time for multiple pollinator visits, resulting in greater pollen donor diversity (Dudash and Ritland, 1991; Karron et al., 2006) and greater overall pollen deposition, both of which can increase pollen competition, depending on the lag between pollen deposition and pollen tube growth (Galen et al., 1986). Considering the large number of ovules per flower in *S. angularis* and gradual pollen deposition rates in the field (R. B. Spigler and S. Ostrowski, unpublished data), we would expect donor diversity per flower to correlate with floral lifespan. Continuous expansion of the stigma lobes could also promote donor diversity or reduce interference from nonviable pollen grains by exposing fresh stigmatic surface (Smith‐Huerta and Vasek, 1984) or simply position stigma lobes so they are more likely to intersect the flight path of floral visitors (Ruan and Teixeira da Silva, 2011). As the *S. angularis* stigma unravels, it also coils. In many species, curvature of styles and stigma lobes decreases the distances to anthers and allows for direct contact, resulting in delayed selfing (e.g., Dole, 1992; Freitas and Sazima, 2009; reviewed by Goodwillie and Weber, 2018), though it does not necessarily serve this function (Goodwillie et al., 2018). Coiling might actually prevent a reduction in herkogamy in *S. angularis* by allowing the stigma lobes to continue to open and expose fresh stigmatic surface without coming into greater contact with anthers. Although we did not explicitly measure herkogamy, we do show that the stigma lobes cease to extend away from the style at the time coiling increases, yet the stigma lobes continue to pull apart and open (measured by the angle of opening) for at least another day (Figure 2). Importantly, stigma/stylar changes may serve more than one function (Ruan and Teixeira da Silva, 2011), highlighting the need to explicitly test multiple hypotheses.

In species capable of autonomous selfing, the schedule of stigma movements and receptivity relative to timing of anther dehiscence in large part dictates the timing of selfing. In between the extremes of prior and delayed, the opportunity for outcross and self‐pollen to arrive at the same time (i.e., competing selfing) can nevertheless span a relatively large range of time in species with appreciably long floral lifespans and will depend on the type and degree of dichogamy (Lloyd and Schoen, 1992). In these cases, the probability of selfing with flower age can vary. For example, the number of pollen tubes resulting from autonomous self‐pollen deposition increased steadily with flower age in *Triodanis perfoliata* (Campanulaceae) (Goodwillie et al., 2018), and there was a near linear increase in autonomous seed production with flower age in the annual gentian *Blackstonia perfoliata* (Brys et al., 2013). Here, we demonstrated that autonomous selfing in *S. angularis* can occur as early as the first day of female phase. Looking at daily mean values (Figure 3B), however, autonomous seed production is low early on and doubles after 6 d (Figure 3D). If we quantify the relative delay of selfing by comparing autonomous seed production relatively early in floral lifespan to that achievable by its end (Lloyd and Schoen, 1992) and consider that *S. angularis* flowers in populations with high pollinator activity live 4–5 d (Dudash, 1991), ~50% of autonomous selfing may be considered competing vs. (relatively) delayed (see Appendix S3). The timing of the increase in autonomous selfing with flower age is likely due to the confluence of anthers accumulating uncollected pollen while stigma lobes continue to unravel. Unraveled lobes present a greater surface area that is more likely to passively capture falling pollen. Indeed, we routinely find fallen pollen on petals when pollinators are excluded (personal observation). Taylor (2016) similarly noticed pollen accumulation on anthers and petals for the congener *S. campetris* but also observed stigmas directly contacting anthers. Though we did not explicitly measure herkogamy, we did not commonly see stigma–anther contact.

### Fitness consequences of competing and delayed pollination and their evolutionary implications

Whereas prior, competing, and delayed selfing can all be advantageous when pollinators are absent because they can provide reproductive assurance, competing selfing in *S. angularis* should be costly when pollinators are abundant due to pollen and seed discounting (Lloyd and Schoen, 1992; Herlihy and Eckert, 2002). Of course, there is an important distinction about what is possible across floral lifespan, demonstrated here, and what actually occurs in wild populations. In wild populations, pollen will only accumulate on the protandrous flowers of *S. angularis* if it is not actively exported, rendering the probability of accumulation, and thus autonomous self‐pollination, dependent on the rate of pollen removal. Pollen export can be rapid in wild *S. angularis* populations, with ~60–75% of pollen removed on average on day 2 and ~85–90% removed by day 3 (Dudash, 1991; R. B. Spigler and S. Ostrowski, unpublished data). Consequently, appreciable levels of autonomous selfing are only likely to be achievable when pollinators are largely absent or ineffective. Nevertheless, prior work has illustrated that these conditions exist in some populations, causing *S. angularis* plants to rely on autonomous selfing for reproductive assurance (Spigler, 2018). Interestingly, we found a significant random population effect on autonomous seed number (Appendix S2), though we cannot determine with the data in hand whether the statistical significance of the random population term is meaningful with respect to variation in population reliance on reproductive assurance in the wild, potentially reflecting past evolution on autonomous selfing. Future work can investigate the extent to which genetically based population variation in autonomous selfing and its timing are connected to herkogamy or dichogamy and either pollination or abiotic conditions in the wild (e.g., Elle et al., 2010; Koski et al., 2018; McElderry et al., 2022).

In considering both the mechanisms of how mating might change with flower age as well as their fitness consequences, we can gain further insight into the evolution and prevalence of mixed mating (Goodwillie et al., 2005; Whitehead et al., 2018). We showed that outcross seed quantity and quality, estimated as seed size, change with flower age, decreasing dramatically after 6 d, while the probability of autonomous seed production increases and seed size remains constant. Similar declines in outcrossed (or naturally pollinated) seed quantity and/or quality with flower age have been shown in a number of other forbs (Webb and Littleton, 1987; Levy, 1988; Jakobsen and Martens, 1994; Petanidou et al., 2001; Arathi et al., 2002; Castro et al., 2008; Marques and Draper, 2012; Hildesheim et al., 2019). Reduced seed production of older flowers under outcross‐pollination could arise for a number of reasons, including stylar senescence and the decline of pollen tube growth rates (Ascher and Peloquin, 1966; Jakobsen and Martens, 1994), declines in ovule viability with flower age (Stösser and Anvari, 1982), or it could represent an indirect cost of floral longevity due to resource‐based trade‐offs (Ashman and Schoen, 1997). Although we cannot rule out changes in resource allocation or pollen tube growth, our data reject declines in stigmatic receptivity. Rather, the linear decline in seed diameter under hand outcross hand pollination with flower age is consistent with reduced ovule viability in older flowers. The net result is a change in autofertility with flower age. In fact, we show autofertility on average occurs triples from early (~0.2) to late (~0.64) floral lifespan. Now, if selfing is delayed or increases across floral lifespan, it is perhaps not surprising that autofertility increases with flower age. However, whereas the probability of autonomous selfing—the numerator in autofertility—may change across floral lifespan, it is rarely appreciated that the denominator—maximum seed production—changes with floral lifespan as well. Our results suggest that this distinction can have important implications. On the one hand, the decline in seed quantity and quality via maximum outcross‐pollination effectively lowers the bar for selfing to be favored when it occurs later in floral lifespan, or at least reduces its costs. On the other hand, seed discounting will be exacerbated under earlier selfing because it coincides with maximum potential seed quality, thus not just squandering ovules that might otherwise have been outcrossed given favorable pollution conditions but also squandering the best quality ovules. Importantly, the change in the cost of seed discounting across floral lifespan should also apply to pollinator‐mediated selfing and could help explain the maintenance of mixed mating (Goodwillie et al., 2005; Devaux et al., 2014). Hildesheim et al. (2019) recently illustrated the interplay between the timing of selfing and floral longevity in the mixed‐mating perennial vine *Dalechampia scandens* (Euphorbiaceae). By considering both changes in flower age and inbreeding depression they estimated that there is an increased cost of delayed selfing in *D. scandens*. One notable difference in their study from ours is that they examined seed production under hand self‐ and outcross‐pollination, akin to high levels of pollinator‐mediated selfing. In the current study, we were concerned with autonomous selfing only. We can rule out inbreeding depression as a cause of the large difference in seed quantity and quality between maximum outcross‐pollination and autonomous self‐pollination seen early in floral lifespan in our study, given prior studies have repeatedly shown similar seed production from flowers hand‐pollinated with either self‐ or outcross pollen (Dudash, 1990; Spigler et al., 2017), even when pollinations are conducted on the first day of female phase (Spigler, 2017).

The steep decline in seed quantity and quality with flower age shown here was surprising given *S. angularis* can live up to ~20 d and begs the question: why live so long? This problem is even more interesting considering a trade‐off between maximum floral longevity and flower number (Spigler and Woodard, 2019). There are several reasons why extended longevity could be maintained. First, many *S. angularis* flowers and those of other species simply never or only rarely reach their maximum potential longevity in wild populations, instead wilting early in response to pollination (van Doorn, 1997). Consequently, opportunities for the expression of and selection on maximum longevity may only occur under extremely poor pollination environments. For example, when pollen is not limiting, *S. angularis* flowers live only ~4–5 d on average (Dudash, 1991; R. B. Spigler, unpublished data), though flowers on plants protected from pollinators in the field have been seen to live as long as ~2 weeks (R. B. Spigler, unpublished data). Pollen‐limited flowers of other species have also been found to live to ages barely able to produce seed (Webb and Littleton, 1987; Castro et al., 2008). Under low pollinator activity, investment in exceptionally long‐lived flowers despite per‐flower fitness declines can nevertheless pay off as form of outcross reproductive assurance (Rathcke, 2003), analogous to potential net gain from delayed autonomous selfing in the face of inbreeding depression. Even a small fitness gain from pollination of old flowers can result in a net benefit provided the cost of flower maintenance is not too great and is lower than the cost of constructing a new flower. Outcross reproductive assurance will be particularly important when pollen removal rates are substantially greater than pollen deposition rates eliminating the option of reliance on autonomous self‐pollination. Field studies confirm that pollen removal and deposition rates are not correlated in at least some *S. angularis* populations (R. B. Spigler and S. Ostrowski, unpublished data), a disconnect seen in other species as well (Wilson and Thomson, 1991). The potential for flowers to live despite diminished seed‐set capacity may also serve a different function, namely, greater plant‐level attractiveness via increased floral display under low pollinator visitation rates (Harder and Johnson, 2005). Indeed, both floral longevity and display size are greater on unpollinated *S. angularis* plants (Spigler, 2017). Our results suggest that even if greater floral display increases the probability of geitonogamy (e.g., Klinkhamer and de Jong, 1993; Snow et al., 1996; Karron et al., 2004), the cost in older flowers can be effectively neutralized while still increasing the chance of outcrossing.

## CONCLUSIONS

Our work demonstrates how developmental changes across floral lifespan can influence pollination dynamics and seed quantity and quality. We found that the ability for autonomous selfing to provide reproductive assurance is not static and instead depends on the relative timing of outcross pollen receipt. In fact, by the end of floral lifespan, seed quantity and quality via autonomous selfing and maximum outcross‐pollination converged, raising questions about the relative importance of reproductive assurance via autonomous selfing vs. outcross reproductive assurance via extended floral longevity. We suggest that declines in seed (or pollen) quantity and quality with flower age be explicitly considered in studies modeling the evolution of floral longevity or timing of selfing and in empirical studies of pollen quantity and quality limitation (Aizen and Harder, 2007). Overall, our work highlights the potential feedbacks between the evolution of floral longevity and the mating system.

## AUTHOR CONTRIBUTIONS

R.B.S. was responsible for conceptualization, funding acquisition, resources, supervision, data validation and visualization, and writing the original draft. R.M. was responsible for performing the experiments and data collection. Both R.B.S. and R.M. contributed to methodology, data curation, and reviewing and editing.

**Table 2:** Mixed‐model results for the fixed effect^a^ of flower age on seed number and size via autonomous selfing and maximum outcross‐pollination across the floral lifespan

	Autonomous selfing												Hand‐pollinated (Outcross)							
	Pollen deposition (log)				Seed number fruit^−1^				Seed size				Seed number fruit^−1^				Seed size			
Flower age	**df**	**Estimate**	***F***	***P***	**df**	**Estimate**	***F***	***P***	**df**	**Estimate**	***F***	***P***	**df**	**Estimate**	***F***	***P***	**df**	**Estimate**	***F***	***P***
Linear	1, 87	0.05	12.18	**0.0008**	1, 256	40.22	14.26	**0.0002**	1, 327	0.001	1.97	0.16	1, 253	22.37	1.11	0.29	1, 195	–0.004	46.17	**<0.0001**
Quadratic					1, 256	–2.36	8.24	**0.004**					1, 259	–3.84	7.91	**0.005**				

^a^ See Appendix S3 for a table of random effects for each model.

## Supporting information


**Appendix S1**. Figure illustrating measurements made on stigmas.

**Appendix S2**. Table comparing the fit of general or generalized linear models including different random effects.

**Appendix S3**. Mean, SE, and SD of trait values, calculated by day of female phase. These values are plotted in Figures 2 and 3 in the main text.

## Data Availability

Data used in the analyses in this work are available on Figshare (https://doi.org/10.6084/m9.figshare.17695427.v1).
