# Supplementary material for: Changes in female function and autonomous selfing across floral lifespan interact to drive variation in the cost of selfing
Source: Am J Bot. 2022 Mar 27;109(4):616–27. doi: 10.1002/ajb2.1816 (PMC9315013; doi:10.1002/ajb2.1816)
Supplement: Supplementary file 1 — Appendix S1. Figure illustrating measurements made on stigmas. [file AJB2-109-616-s002.docx]

Spigler & Maguiña—American Journal of Botany 2022 – Appendix S1

**Appendix S1**. Figure illustrating measurements made on stigmas.


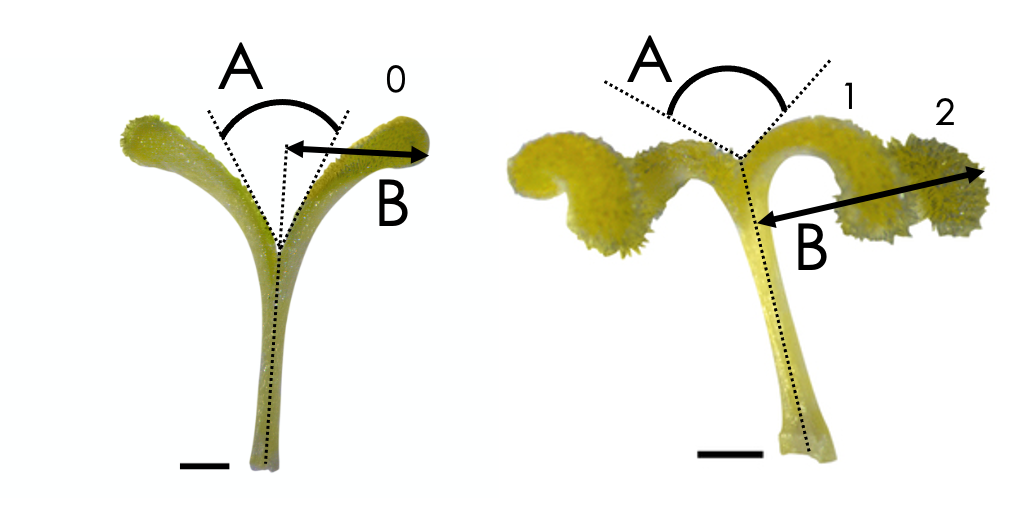


Measurements made on stigmas across floral lifespan. Two examples are given representing day 2 (left) and day 12 (right). A = the angle of opening between the stigma lobes, based on a central line parallel to the style and the line tangent to the stigmatic surface. B = stigma lobe extension, measured as the distance from the style to the tip of the stigma at an angle perpendicular to the style. In both cases, measurements were made on each lobe and the average used in analyses. The number of twists on each picture is indicated (0, 1, 2). Scale bar in each photo is 1mm.
