# Supplementary material for: Changes in female function and autonomous selfing across floral lifespan interact to drive variation in the cost of selfing
Source: Am J Bot. 2022 Mar 27;109(4):616–27. doi: 10.1002/ajb2.1816 (PMC9315013; doi:10.1002/ajb2.1816)
Supplement: Supplementary file 2 — Appendix S2. Table comparing the fit of general or generalized linear models including different random effects. [file AJB2-109-616-s003.docx]

Spigler & Maguiña—American Journal of Botany 2022 – Appendix S2

**Appendix S2**. Table comparing the fit of general or generalized linear models including different random effects for each trait.

^1^We tested hierarchical models. In cases where there was more than one top model ($\Delta$AIC<2), we chose the more parsimonious model (indicated in boldface)

^2^For some traits model complexity precluded testing for random population effects.

^3^Based on plots of the raw data and residuals, for some traits we also included a grouping factor in proc mixed or glimmix to account for heterogeneity of variances among populations.
