# Supplementary material for: Changes in female function and autonomous selfing across floral lifespan interact to drive variation in the cost of selfing
Source: Am J Bot. 2022 Mar 27;109(4):616–27. doi: 10.1002/ajb2.1816 (PMC9315013; doi:10.1002/ajb2.1816)
Supplement: Supplementary file 3 — Appendix S3. Mean, SE, and SD of trait values, calculated by day of female phase. These values are plotted in Figures 2 and 3 in the main text. [file AJB2-109-616-s001.docx]

Spigler & Maguiña—American Journal of Botany 2022 – Appendix S3

**Appendix S3**. Mean, SE, and SD of trait values, calculated by day of female phase. These values are plotted in Figures 2 & 3 in the main text.
